# Immune exhaustion in esophageal cancer: interferon pathway dysregulation and neoadjuvant therapy response

**DOI:** 10.3389/fcell.2026.1898443

**Published:** 2026-07-30

**Authors:** Xiaoshuang Wu, Shegan Gao, Yijun Qi

**Affiliations:** State Key Laboratory of Esophageal Cancer Prevention and Treatment, Henan Key Laboratory of Microbiome and Esophageal Cancer Prevention and Treatment, Henan Key Laboratory of Cancer Epigenetics, Cancer Hospital, The First Affiliated Hospital, College of Clinical Medicine, Medical College of Henan University of Science and Technology, Luoyang, Henan, China

**Keywords:** biomarker, esophageal cancer, immune exhaustion, neoadjuvant therapy, tumor immunogenicity interferon

## Abstract

Esophageal cancer (EC) is an aggressive malignancy with poor survival despite advances in multimodal treatment. Local control of the disease is possible with chemotherapeutic, radiotherapeutic, and chemoradiotherapeutic neoadjuvant therapies, although treatment resistance and recurrence are common, and disease progression continues. Several studies show that the so-called immune exhaustion in the tumor microenvironment (TME) is the most significant cause of failure of the aforementioned treatments. Specifically, dysregulated interferon (IFN) signaling may have paradoxical effects in EC, promoting antitumor immune activation in some contexts while contributing to immune suppression, checkpoint upregulation, and tumor adaptation when chronically activated. This review examines whether IFN pathway dysregulation, particularly involving IER2 and IFNGR1, may contribute to immune exhaustion and influence neoadjuvant therapy response. In particular, we examine the role of the interferon-related genes (IRGs) in the immediate early response 2 (IER2) and interferon gamma receptor 1 (IFNGR1) in the mechanistic clinical role of neoadjuvant therapies. It includes radiotherapy, chemotherapy, and immune remodeling. Evidence of persistent IFN signaling T cell exhaustion, immune checkpoint upregulation, the phenomenon of adaptive resistance and the lack of therapeutically adequate sustained efficacy are analyzed. Finally, we show how the changes in the tumor immune microenvironment (TIME) caused by the neoadjuvant therapy (NAT) induced IFN activation. In particular, we examine the changes in the cross-presentation of immunogenic cells, cytokines, cytokine receptors, and exhaustion. This paper outlines a structure connecting IFN-induced immune exhaustion with responses to neoadjuvant therapy in EC, integrating epidemiology, molecular crossroads, and innovative translational research. To conclude, we examine prospective treatment efforts to address immune exhaustion through IFN pathway adjustment, strategic combination therapies, and biomarkers to refine patient stratification. A clearer understanding of context-dependent IFN signaling may support biomarker-guided neoadjuvant strategies and rational combination therapies in EC.

## 1 Introduction

Esophageal cancer (EC) is one of the most fatal forms of cancer globally, with over 500,000 annual deaths worldwide (Sheikh et al., 2023). It is the ninth most common cancer and the sixth leading cause of death. Esophageal squamous cell carcinoma (ESCC) and esophageal adenocarcinoma (EAC) are two main subtypes with variable clinical and epidemiological behaviors, with <20% 5-year survival rate (Uhlenhopp et al., 2020). Because ESCC and EAC differ in epidemiology, molecular drivers, immune landscape, and therapeutic response, evidence should be interpreted according to histological subtype whenever available. Throughout this review, findings are described as ESCC-specific, EAC-specific, or pan-EC when the cited evidence permits such distinction. EC is more common in men than women, known for its aggressive behavior, late-stage diagnosis, and poor prognosis (Sung et al., 2025). Despite the development of various treatment approaches, survival rates post-surgery remain exceedingly low (Waters and Reznik, 2022). Recently, the focus of oncology has shifted to the tumor immune microenvironment (TIME) as a crucial factor in patient response to treatment (Liu et al., 2025). Of particular interest to researchers is immune exhaustion, which explains patient resistance to most therapies and the ineffective treatment response to new immunotherapies (Wan et al., 2026). To increase patient treatment outcomes in EC, the molecular and immunological mechanisms that cause immune exhaustion must be elucidated (Turki et al., 2025). This article is a narrative review. Relevant literature was identified from published peer-reviewed articles and publicly available clinical trial information focusing on esophageal cancer, tumor immune microenvironment, interferon signaling, immune exhaustion, DNA damage response, and neoadjuvant therapy. Priority was given to studies directly involving esophageal squamous cell carcinoma or esophageal adenocarcinoma. Where evidence was derived from other cancer types or preclinical models, this has been stated explicitly. This review does not present unpublished data, original patient-level data, or a systematic meta-analysis.

### 1.1 Clinical burden and therapeutic challenges of EC

EC is a significant global health issue, constituting one of the most deadly forms of cancer (Sung et al., 2021). EC disproportionately affects different regions and populations, with ESCC highly common in East Africa and Asia, whereas in Western populations, EAC is also common (Sung et al., 2021; Xia et al., 2022). Regardless of the type, most patients present with late-stage, locally-advanced, or metastatic diseases that may not be surgically resectable (Baek et al., 2024). For resectable locally advanced EC, neoadjuvant treatment strategies, which include chemotherapy, radiotherapy, and chemoradiotherapy, have been incorporated into the standard of care (Miller et al., 2026). These strategies are used to reduce tumor size, eliminate micrometastatic disease, and improve surgical outcomes. Nevertheless, clinical outcomes from neoadjuvant therapy (NAT) vary greatly and are often poor (Chen B. et al., 2026). While a subgroup of patients experience a pathological complete response and improve their prognosis. The majority of patients experience low response or complete resistance and experience disease recurrence and progression (Wan et al., 2026). The inability of NAT to provide long-term benefit exposes the intricate adaptive patterns of the tumor microenvironment (TME) (Baek et al., 2024). Alongside tumor-intrinsic genetic alterations, emerging data indicate that treatment can remodel specific immune signaling pathways, favoring selective or nonselective therapeutic outcomes (Wu et al., 2026). Because ESCC and EAC differ substantially in their molecular drivers, immune microenvironment composition, and therapeutic sensitivities, evidence in this review is described as ESCC-specific, EAC-specific, or derived from mixed EC cohorts wherever the cited literature permits such distinction. Interferons (IFN) have been classically viewed as a key component of the immune system involved in fighting the tumor (Garcia-Diaz et al., 2017). In this regard, receptor-induced signaling is paradoxically tumor-promoting and immunosuppressive following cytotoxic therapy (Fasquelle et al., 2026).

### 1.2 Immune exhaustion as a mechanism of treatment failure

Chronic exposure to a specific antigen and excessive inflammatory signal leads to a condition known as immunological exhaustion (Wang et al., 2025). From an immunological standpoint, exhaustion occurs when there is a state of progressive dysfunction in cytotoxic T lymphocytes (Lai N. et al., 2025). Exhausted immune cells tend to have a lower capacity to proliferate, to produce cytokines, and to express more of the so-called immune checkpoint receptors (ICRs) such as PD-1, CTLA-4, TIM-3, and LAG-3 (Lin et al., 2024; Cui et al., 2025; Yang et al., 2022). In EC, immune exhaustion is a central categorization to be ascribed to the TIME, and thus, an important contributor to the widening gap in antitumor immune responses (Liu et al., 2025). Immunological neoadjuvant chemotherapy and radiotherapy are the only clinically approved treatment modalities, acting as a double-edged sword (Chen B. et al., 2026). Despite their prolonged immunological effects, these drive immunological exhaustion and fall within the realm of chronic persistent immune stimulation. In these instances, it is particularly applicable with respect to the signal transduction pathways of interferon (IFN), as their immune regulatory variables are contextually oriented (Lai N. et al., 2025). In instances where the activation of the IFN pathway is of an acute, short-term, or temporally confined nature, regulatory T cells are inhibited, tumor antigen presentation is enhanced, and T cells are primed (Arimoto et al., 2018; Cheng et al., 2026). Conversely, when there is chronic or prolonged exposure to an immune regulatory signal induced by IFN, and ICRs are expressed, the regulation of the immune system by the immune regulatory cells is augmented, and immune evasion of the tumor is promoted (Lai X. et al., 2025).

**FIGURE 1:**
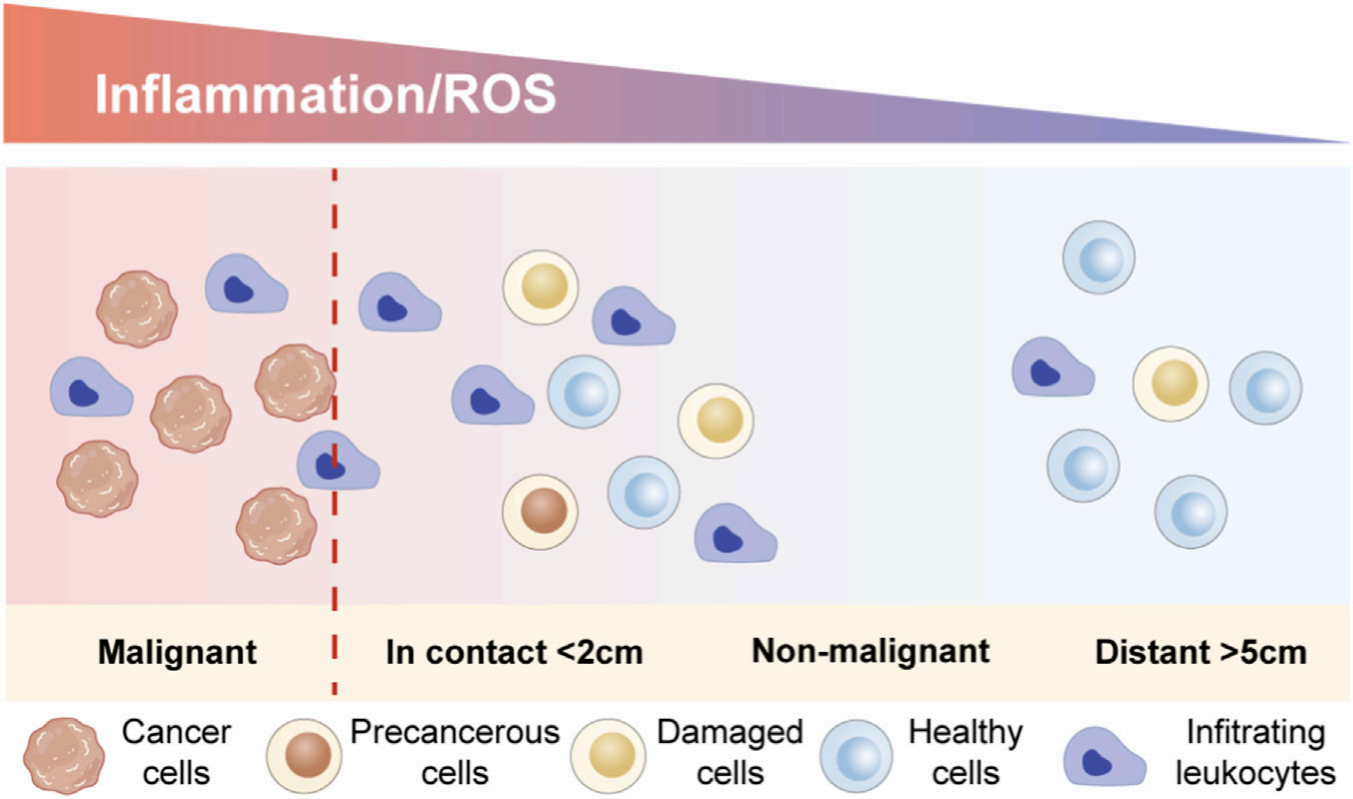
Esophageal cancer progression model (He et al., 2013). The EC cells and infiltrating leukocytes generate ROS and damaging inflammatory species to trigger DDR in the tumor-surrounding non-malignant tissues, thus enhancing tumor progression.

It has been indicated that dysregulated components of the IFN pathway operate not only as immune mediators but also as resistance-associated factors influencing the shaping of therapeutic response (Han et al., 2025). Immediate early response 2 (IER2) and interferon gamma receptor 1 (IFNGR1), for example, have been implicated in the maintenance of IFN-driven signaling loops that sustain immune exhaustion and adaptive resistance to radiotherapy and chemotherapy (Zhang B. et al., 2023). Therefore, immune exhaustion is a key central mechanistic link between the NAT, activation of the IFN pathway, and the treatment failure of EC (Wu et al., 2026). Overall, the cases emphasize immune exhaustion as an important factor for the therapeutic and clinical success of the intervention, and as a research target of significant merit (Chen B. et al., 2026). The sufficient molecular understanding of the networks of immune exhaustion that are provoked by the IFN pathway components is likely to facilitate the creation of rational combination strategies to optimize the NAT and prolong survival in EC (Hoeppner and Nimczewski, 2025). A model has been proposed wherein cancer cells generate damaging factors like inflammatory cytokines into the surrounding environment, which, along with infiltrating leukocytes in the TME, cause inflammation and ROS production (Figure 1) (Wen et al., 2024). These negative signals trigger DNA damage and DDR, which may contribute to the tumor commencement and development (He et al., 2013). Another hallmark of tumorogenesis in ESCC is hypoxia, and recently, a hypoxia-induced long non-coding RNA (lnc191) is positively expressed in patients with poor prognosis. Hypoxia-inducible factor-1α (HIF-1α) is involved in the mediation of transcriptional activation of lnc191, which increases tumor growth and metastasis both during *in vitro* and *in vivo* assays by the activation of GRP78/ERK Pathway (Wei et al., 2025). In another study, hypoxia inhibited the iMo/cDC2/CD8+TRMs immune axis in the TME of human EC (Wu et al., 2024). On the other hand, it has been shown that hypoxia triggers chemoresistance against cisplatin-treated EC via the augmentation of IncRNA-EMS/miR-758–3p/WTAP axis (Zhu et al., 2021). The work on the role and mechanism of HIF-1α and chemoresistance in EC is continued for the determination of a therapeutic target (Sun, 2026). Throughout this review, we adopt a central conceptual framework: acute and temporally limited IFN pathway activation supports antitumor immunity by enhancing antigen presentation, T cell priming, and innate immune effector functions. In contrast, chronic or sustained IFN signaling—as occurs during prolonged neoadjuvant treatment—promotes immune checkpoint upregulation, exhausted T cell phenotypes, and tumor immune adaptation.

## 2 Epidemiology and etiological factors of EC

The complexities of ecology, environmental exposure, lifestyle phenomena, host genome, and inflammation response paint a broad picture of the geographic disparity seen in EC, along with the incidence, histological variety, and causative factors (Choday et al., 2025). The added variability of the disease and geographic diversity of the immune framework, along with the varied response to treatment among the etiological and epidemiological differences, explain the cause of the immune landscape and therapeutic response (He and Ke, 2025).

### 2.1 Epidemiological data

EC ranks in the top six of the deadliest cancers in the world (Li et al., 2021). The high mortality rates are largely due to the high incidence in the greater East Asia, Central Asia, and Eastern and Southern Africa (Sung et al., 2021; Bray et al., 2018). The greatest concentration of these deaths by deaths to incidence ratio is found in the ESCC of the eastern ‘esophageal cancer belt’ (Wang B. et al., 2022). Conversely, the West has seen rapid increases in the incidence of EAC, largely due to lifestyle changes and the epidemics of reflux disease and obesity (Kaur et al., 2025; Siegel et al., 2023). The collection of diverse deaths in the world’s epidemiological data suggests there is a strong need for a new path for the analysis of international cancer mortality data and designing regionally specific cancer control programs for the said countries (Boshier et al., 2026).

### 2.2 Risk factors and disease heterogeneity

The onset of EC has many causes and greatly varies depending on the subtype of cancer (Li et al., 2021). EC risk factors include tobacco smoking, overconsumption of alcohol, opium, dietary nitrosamines, a lack of sufficient vitamins and minerals, and thermal injury from the consumption of very hot beverages (Kaur et al., 2025). The socioeconomic status, the type of household fuel used, contamination from mycotoxins and contact with animals may contribute towards ESCC risk factors. Poor oral hygiene, diversity of oral and esophageal microbiome, quality of food intake, poor quality water and micronutrients also contribute towards risk assessment in the development of EC (Lander et al., 2023). There are inconsistent associations with viral and bacterial infections, whereas *Helicobacter pylori* has shown an inverse association with EAC, 50% risk decrease, and Treg cells have been suggested as targets for such a population of patients (Sheikh et al., 2023; Matsuda et al., 2024). Chronic exposure to the previously mentioned risk factors leads to epithelial damage, instability, inflammation, and the eventual malignant transformation of cancer. On the other hand, EAC has risk factors that include gastroesophageal reflux disease (GERD), Barrett’s esophagus, obesity, and metabolic dysregulation (Sung et al., 2025). GERD is the primary EAC risk factor. The reflux causes damage to the esophagus that leads to the metaplastic and dysplastic changes. It results in the inflammation of the epithelial lining, which leads to the remodeling of the epithelium (McLaughlin et al., 2020).

EAC and ESCC differ at the molecular levels of cancer (Yang et al., 2020). The genetic studies have shown differential mutational patterns between ESCC and EAC. The molecular signatures of ESCC have shown mutations classified into three groups: ESCC1 (50), ESCC2 (36) and ESCC3 (4) (Cancer Genome Atlas Research Network et al., 2017). These mutations were responsible for modifications in NRF2, autophagy, Hippo, cell cycle regulation, Notch, RTK-MAPK-PI3K and Wnt pathways (Sasaki et al., 2016). A small number of mutations are common between ESCC and EAC genetic signatures, with TP53 being common between them. Mutations were defined in two subgroups of subtype I (24 genes) and subtype II (30 genes) (Cancer Genome Atlas Research Network et al., 2017; Dulak et al., 2013). For example, ESCC has unique signaling dependencies and is lacking in many of the immune cells found in EAC, with significant inter and intra-tumor heterogeneity (Cui et al., 2025; Jing et al., 2022). Factors such as interferon signaling, immune regulatory molecules, and other defenses contribute to the heterogeneity (Wei et al., 2019). Tumors that have more extreme forms of immunosuppression or immune exhaustion are unlikely to respond to NATs or other immune-modulating treatments (Akumaga et al., 2025).

### 2.3 Role of microbiota and chronic inflammation

Chronic inflammation is a unifying cause of esophageal carcinogenesis. Inflammation, environmental exposures, and immune dysregulation are also mutually interlinked (Zhou et al., 2026). In addition, tumor evolution is also linked to the same trio. Persistent inflammatory stimuli drive continuous immune activation, which over time can lead to immune exhaustion (Wang et al., 2018). The esophageal and upper gastrointestinal microbiota have emerged as important modulators of local inflammation and carcinogenic risk (Xiong et al., 2025). The enrichment of proinflammatory microbial species has been linked to both ESCC and EAC diseases (Zhang and Li, 2026). Injurious dysbiosis activates epithelial injury, and also activates pattern recognition receptors and stimulates the TIME (Liu et al., 2025). Persistent dysbiosis enhances immune surveillance and also favors immune exhaustion, leading to immune escape and increasing tumor progression (Klimov-Kravtchenko et al., 2025). Microbiota-driven inflammation has been linked to modulated responses to chemotherapy and also to radiotherapy (Huang et al., 2024). These findings suggest that microbial and inflammatory factors are important to consider within the context of EC (Houle and Abrams, 2026). It also indicates that microbial and inflammatory factors can serve to better stratify patients and design clinically targeted interventions (Xie et al., 2026). The patterns and chronic inflammation aligned with immune dysregulation are cardinal to EC. Understanding how the factors contribute to immune exhaustion is essential for recognizing the differences in responses to NAT, as well as for devising improved tailored treatment approaches (He et al., 2019).

## 3 Tumor immune microenvironment in EC

**FIGURE 2:**
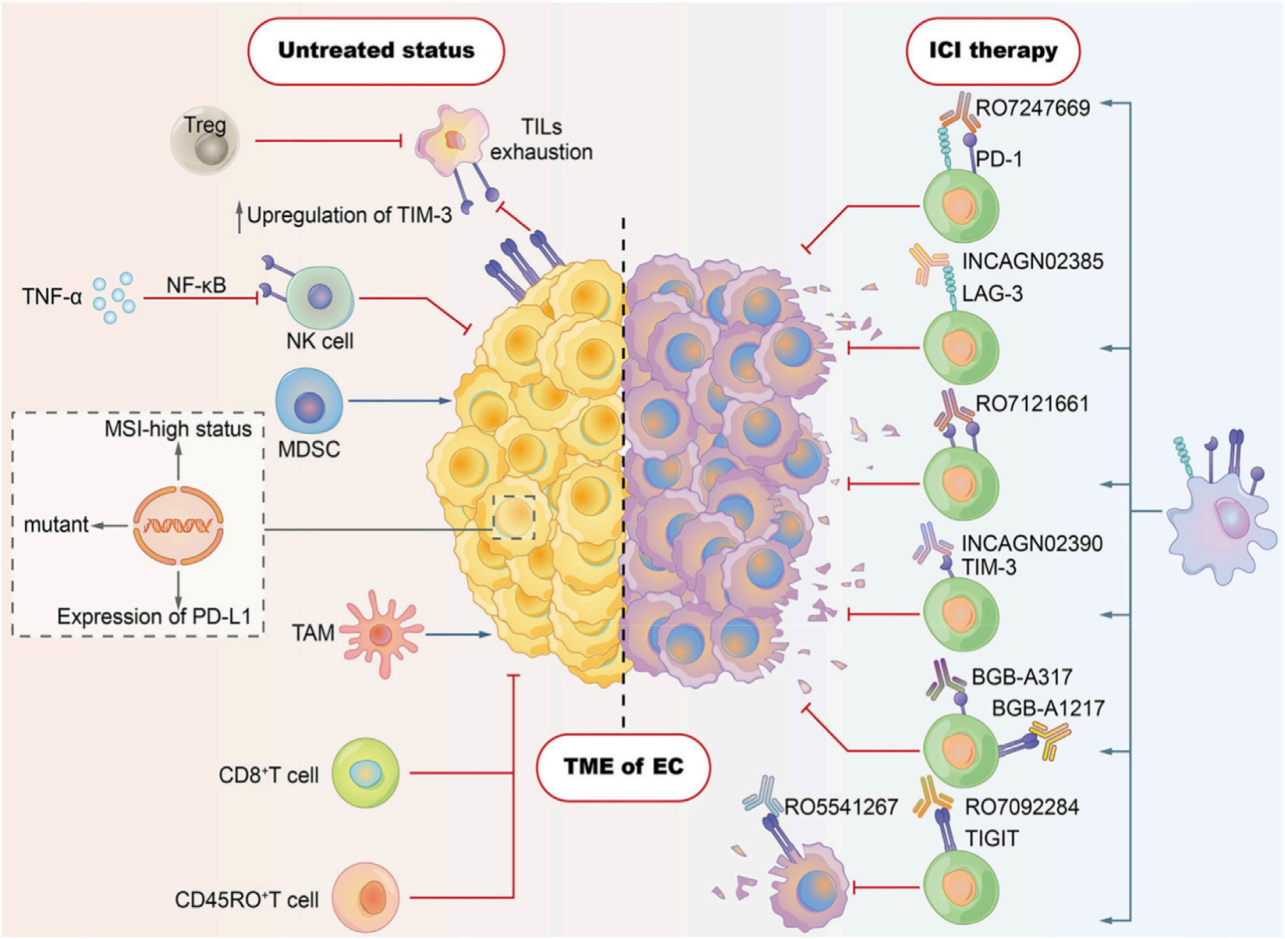
TME in esophageal cancer (Zhou et al., 2022). Tumor cells escape the host immune system through several extrinsic factors comprising the TME. ICIs reverse the immunosuppressive effects and enhance immune response against tumors. In untreated cases, CD8^+^ T cells, CD45RO + T cells, and NK cells mediate the antitumor response, while others possess suppressing effects. Tregs diminish the activity of TILs. TAMs and MDSCs enhance the immune evasion. The intrinsic factors like PD-L1, mutation load, and MSI-high status confer resistance to ICIs (TIGIT, PD-1), linked to TIL exhaustion. The NK cells are attributed to TNF-α and stimulate TIM-3 expression via the NF-κB signalling pathway. On the other hand, ICIs target LAG-3, TIM-3, and TIGIT, aiming to reverse the exhaustion or dysfunction states of T cells, as shown in the right section of the figure. Reprinted from Zhou X, Ren T, Zan H, Hua C and Guo X (2022) Novel Immune Checkpoints in Esophageal Cancer: From Biomarkers to Therapeutic Targets. Front. Immunol. 13:864,202. Doi: 10.3389/fimmu.2022.864202.

The TIME in EC are constantly changing, in large part due to intrinsic tumor changes, chronic inflammation, and therapy-related changes (Liu et al., 2025). Instead of creating a durable and successful antitumor response, immune cell infiltration in EC tends to shift to a more unproductive and immunosuppressive pattern (McLaughlin et al., 2020). This immune response uncoupling is a key driver of development, immune evasion, and resistance to NAT (Figure 2) (Zhou et al., 2022; Hsiehchen et al., 2024). Four key immune cells have been linked with the prognosis of EC patients using the gene set variation analysis method, which comprised NK T cells, immature B cells, NK cells and type 1 T helper cells. The TMEscore was found to be a reliable indicator of the prediction of data outcome of EC patients (Zhang et al., 2021). Recently, a TIME atlas of EC has been reported by augmenting single-cell RNA sequencing and bulk RNA sequencing data, which determines the composition, developmental differentiation, functions and communication between cells in the EC microenvironment. The data was used in the development of a prognostic model and validated risk scores among low and high-risk groups, which are important in the prognosis. The data may be used in the prediction of biomarkers for maximal benefits of immunotherapy (Wen et al., 2024).

### 3.1 Cellular infiltration and functional exhaustion

EC is generally accompanied by the presence of different types of immune cells, such as cytotoxic CD8^+^ T cells, CD4^+^ helper T cells, Treg cells, TAMs, dendritic cells, and MDSCs (Cui et al., 2025; Tseng et al., 2025; Tan et al., 2026; Zhang et al., 2025). In certain patient subgroups, elevated levels of immune infiltration have been correlated with better outcomes (Hsiehchen et al., 2024). However, the presence of higher numbers of immune cells is not always indicative of functional activity. Tumor-infiltrating lymphocytes show symptoms of functional exhaustion (Mai et al., 2026). These symptoms include active cell death, decreased levels of cytokines produced, and a reduction in the cell proliferation rate. This type of exhaustion is predominantly caused by persistent, chronic inflammation signaling, and repeated stimulation with tumor antigens (McLaughlin et al., 2020). Tregs and M2-polarized macrophages, together with other immunosuppressive cell types, gather in the TME and prevent any antitumor immunity effects (Tan et al., 2026). It is vital that neoadjuvant chemotherapy and radiotherapy can lead to a brief increase in immune infiltration due to immunogenic cell death (ICD) and antigen release (Hsiehchen et al., 2024). Post-NAT analyses have shown increased gene expression related to myeloid cell differentiation, alongside a weak prognostic profile associated with high neutrophil CD15^+^ counts (Cabalag et al., 2023). The synergistic anti-tumor effect has been exerted by this combination of immunotherapy and radiotherapy. Radiotherapy triggered and augmented autophagy in cancer and immune cells, which affected the TIME landscape (Zhou and Yang, 2025). However, without a proper strategy to boost the immune system, such an influx will most likely strengthen exhaustion programs and result in the persistence of the tumor and adaptive resistance (Wan et al., 2026).

### 3.2 Immune checkpoint expression and T Cell dysfunction

**FIGURE 3:**
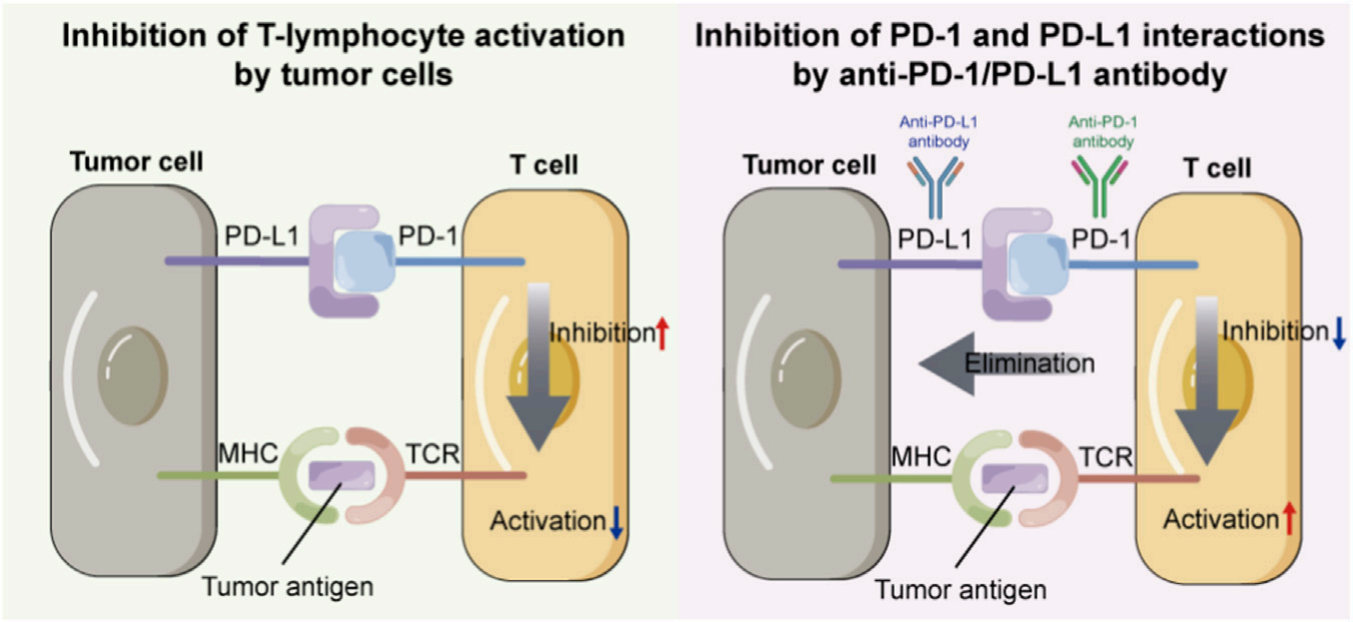
Mechanism of immune checkpoints and their blockade (Nagat et al., 2022). PD-1 is expressed on T lymphocytes and its ligands, PD-L1 and PD-L2, are expressed on the surface of tumor cells. Overexpression of PD-L1 results in tumor progression. Tumor cells use the PD-1/PD-L1 and PD-1/PD-L2 pathways to promote an immunosuppressive environment. ICIs like anti-PD-1 and anti-PD-L1 antibodies inhibit the PD-1/PD-L1 and PD-1/PD-L2 pathways.

A prime example of immune exhaustion in EC is the overexpression of inhibitory immune checkpoints on T cells that have infiltrated tumors. Elevated expression of PD-1, CTLA-4, TIM-3, and LAG-3 has been reported in ESCC (Lin et al., 2024; Cui et al., 2025; Yang et al., 2022); comparable systematic data in EAC are more limited, though similar checkpoint upregulation patterns have been described in mixed EC cohorts. It is important to note that upregulation of PD-1 alone does not confirm T-cell exhaustion. PD-1 is physiologically induced upon T-cell activation and may reflect an antitumor response rather than dysfunction. Functional exhaustion is more rigorously defined by the co-expression of multiple inhibitory receptors (PD-1, TIM-3, LAG-3, TIGIT), progressive loss of effector cytokine secretion (IFN-γ, TNF-α, IL-2), impaired proliferative capacity, and a characteristic transcriptional program driven by TOX and TOX2. Studies in this review that report checkpoint upregulation without accompanying functional T-cell data are interpreted accordingly with appropriate caution. T cell checkpoint engagement inhibits the T cell receptor (TCR) signaling and diminishes the functions of T cells. Immune checkpoint expression is subject to the influence of declining cytokine levels and therapeutic stress, and in a quasi-static state (Nagat et al., 2022). It has been reported that higher plasma fibrinogen levels showed poor prognosis and diminished ICI efficiency in ESCC patients (Ho et al., 2026). Treatment by radiotherapy and chemotherapy can cause PD-L1 expression to increase via interferon (Miller et al., 2026; Yi et al., 2021). It, in turn, increases inhibitory signaling and contributes to the development of resistance to therapy (Figure 3) (Zhang B. et al., 2023). The immune landscape, dominated by immune checkpoints, provides a mechanistic rationale for the use of immune checkpoint blockade (ICB) in EC (Topalian et al., 2023). However, the responses to immunotherapy in the clinic are heterogeneous, which indicates that checkpoint inhibition is net suboptimal if the fundamental drivers of immune exhaustion remain unresolved, such as chronic hypersecretion of INF and immune-oncologic alteration (Wan et al., 2026; Pointer et al., 2022; Galluzzi et al., 2023). The immune checkpoint expression and coexpression have been displayed in several classes of cancers, including melanoma, non-small cell lung cancer and gastroesophageal adenocarcinoma (Vedire et al., 2024). The application of immune checkpoint inhibitors (ICIs) and PD-1 antibodies is revolutionizing EC treatment as several clinical trials are showing promising results, which are intended to improve the prognosis in the future. The details of the outcomes of the clinical trials and applications have been reviewed (Nagat et al., 2022).

### 3.3 Cytokine imbalance and interferon signaling dysregulation

Cytokine profiles determine the immune microenvironment of EC, and a persistent imbalance between pro-inflammatory and immunosuppressive cytokines leads to immune dysfunction and tumor progression (McLaughlin et al., 2020). This paradoxical immune outcome is shaped by the concurrent activity of immunosuppressive mediators such as TGF-β, IL-10, and VEGF, which exist with enduring activation of inflammatory pathways, thus producing a paradoxical immune response (Shimabukuro-Vornhagen et al., 2012). Acute activation of IFN is beneficial, as it enhances antigen signaling and elicits an antitumor response. In contrast, persistent IFN signaling promotes inhibitory checkpoint expression, T-cell exhaustion, and adaptive tumor responses (Pang et al., 2025) (Garcia-Diaz et al., 2017). Imbalanced IFN signaling in EC is correlated with the unresponsiveness of cytotoxic therapies and the mechanisms of immune escape. Recently, the role of certain components, specifically IFN pathways, was considered to have roles aligning with the tumor function, creating and perpetuating chronic IFN signaling and reinforcing immune exhaustion (Lai X. et al., 2025). This IFN-driven state reprograms the immune cells in the microenvironment and creates immune and tumour cell microenvironments that favor therapeutic stress. The imbalance of interferon signaling alludes to the immune exhaustion, the response to therapy of EC, and the progression of the disease (Gao and Sun, 2026). In a phase II trial, sotigalimab, an agonistic anti-CD40 mAb, combined with neoadjuvant chemoradiation has been investigated. Results have shown that sotigalimab decreased the frequency of Tregs in the TME with effective immune responses and improved clinical performance (Soto et al., 2024).

## 4 Interferon signaling pathway: dual roles in cancer

The IFN signaling pathways have both positive and negative impacts on EC (Garcia-Diaz et al., 2017). On the positive side, IFN leads to the stimulation of both the innate and the adaptive arms of immunity in the body, particularly focusing on tumor targeting, immune system activation, and response to therapy. On the negative side, IFN pathways lead to immune redirection and dysregulation of the system (Gao and Sun, 2026). They lead to immune system fatigue and the adaptation of tumor cells so that they become resistant to therapy. This duality poses a fundamental paradox in cancer therapy. Throughout this review, we adopt the following conceptual framework: acute and temporally limited IFN activation supports antitumor immunity by enhancing antigen presentation, T-cell priming, and innate effector functions; chronic or sustained IFN activation—as occurs under prolonged NAT—drives immune checkpoint upregulation, T-cell exhaustion, and tumor immune adaptation. The transition between these two states is governed by the duration and intensity of IFN signaling, the tissue context, and the activity of IFN-pathway components such as IER2 and IFNGR1. This framework is applied consistently throughout the subsequent sections (Topalian et al., 2023).

### 4.1 Canonical IFN signaling and antitumor immunity

Type I (IFN-α/β) and Type II (IFN-γ) interferons, along with their receptors, activate the JAK-STAT pathway (Garcia-Diaz et al., 2017). Canonical IFN signaling starts with the engagement of one of these receptors. An important process in this pathway is the transcription of interferon-stimulated genes (ISGs), which increase the recruitment of immune cells and catalyze stronger immune responses (Gao and Sun, 2026). In antitumor immunity, IFN signaling further drives activating IFN expression and cross-presentation by dendritic cells. It improves IFN signaling to T and natural killer (NK) cells, which also expands their effector and cytolytic functions (Cui et al., 2025). This is the central reasoning for enhancing immune responses to non-immunogenic tumor cells, for the synergistic effect with ICB, and for the increase of responsiveness to ICB in chemotherapy and radiotherapy. These functions help integrate IFN signaling into cancer therapy and early responses to therapy (Topalian et al., 2023).

### 4.2 IFN-driven immune exhaustion and tumor adaptation

Although IFN signaling is essential for antigen presentation, innate immune activation, T-cell priming, and early antitumor immunity, chronic or sustained IFN pathway activation can induce negative immune adaptations that erode long-term antitumor responses (Moughnyeh et al., 2024). This transition from immune activation to immune dysfunction highlights the context-dependent nature of IFN signaling in cancer therapy. T-cell exhaustion occurs with the steady state expression of immune checkpoints, such as PD-1 and PD-L1, and other inhibitory receptors due to chronic IFN exposure (Lin et al., 2024; Cui et al., 2025; Yang et al., 2022; Yi et al., 2021). Exhausted T cells lose the capability to secrete critical cytokines, become functionally deficient, and can lose the ability to proliferate, which ultimately limits the aptitude of these cells to control the growth of tumors (Peng et al., 2023). Tumor cells adapt to immune and therapy pressures by utilizing chronic IFN signaling. Activated, persistent ISGs can promote immune evasion and metabolic adaptations to survive under lethal conditions (Gao and Sun, 2026). In EC, IFN-related transcriptional programs are linked to poor outcomes with chemotherapy and radiotherapy, indicating that IFN signaling might influence the development of adaptive resistance mechanisms after NAT (Wu et al., 2026). Moreover, several studies document that certain IFN pathway components can display properties of an oncogene, maintaining an autonomous signaling loop for immune suppression. This phenomenon, from the immune activation and failure to exhaustion, emphasises the signaling pathways of IFN. Its contradictory effects can reinforce the importance of the context-dependent impact on tumor growth and treatment regimens (Gao and Sun, 2026).

### 4.3 Crosstalk between IFN signaling and DNA damage response

**FIGURE 4:**
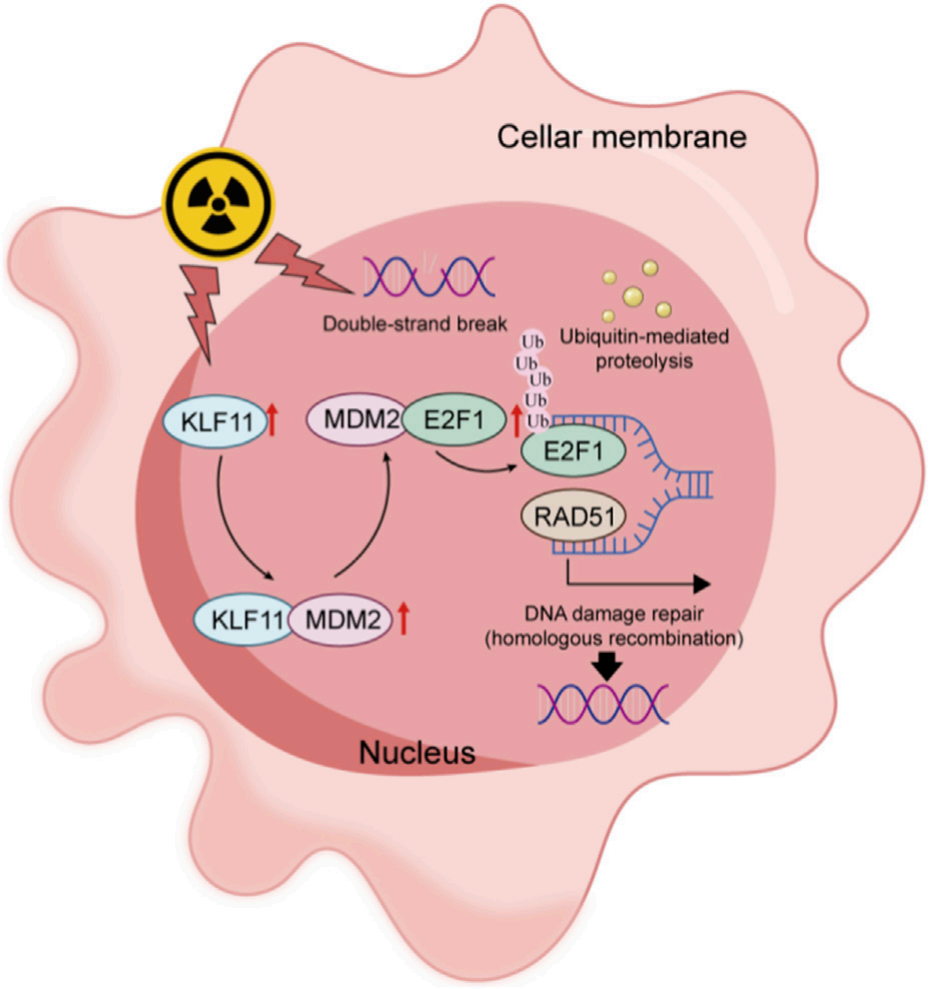
Proposed crosstalk between radiotherapy-induced DNA damage response and IFN signaling in esophageal cancer. The figure summarizes how DNA damage may activate cGAS–STING signaling, induce IFN production, and contribute either to antitumor immune activation or, when sustained, to immune exhaustion and adaptive resistance (Xia et al., 2026). Adapted with permission from Xia Y, Wang J, Zhao D, Li Y, Li S, Li M, Dong R, Wang L. KLF11 interacts with MDM2 to stabilize E2F1 and promotes DNA damage repair to induce radioresistance in esophageal cancer cells. Pathol Res Pract. 2026 April; 280:156375. Doi: 10.1016/j.prp.2026.156375.

Perhaps one of the most intriguing and under-appreciated dimensions of IFN biology in cancer is its cross-talk with the DNA damage response (DDR) (Ceccaldi and Cejka, 2025). Some of the most common cancer treatments, such as radiotherapy and several types of chemotherapy, cause damage to the DNA (Xia et al., 2026). The formation of cytosolic DNA fragments may activate innate immune sensors like cGAS and STING (Kwon and Bakhoum, 2020). Activation of the cGAS–STING pathway can induce type I IFN production, linking genotoxic stress with innate immune activation (He et al., 2013; Li et al., 2026). However, the opposite can happen, and the sustained signaling of the DDR can cause a persistent IFN production, which can lead to immune exhaustion and adaptation by the tumor (Figure 4) (Xia et al., 2026). If tumor cells have defective or altered DDR pathways, they can use the IFN signaling pathways to survive the genotoxic stress, bypass immune surveillance, and gain resistance to NAT (Hoeppner and Nimczewski, 2025). In EC, which relies a great deal on the radiotherapy-induced DNA damage, the IFN signaling pathways both have the critical potential balance to be beneficial as well as harmful (Xia et al., 2026; Kwon and Bakhoum, 2020). NAT may therefore produce divergent immune outcomes: effective immune-mediated tumor control in some patients, or chronic IFN–DDR signaling, immune exhaustion, and adaptive resistance in others (Ceccaldi and Cejka, 2025).

## 5 IFN pathway components with potential oncogenic or resistance-associated roles in EC

IER2 and IFNGR1 are highlighted in this review because they represent distinct but potentially complementary aspects of IFN-related immune regulation: IER2 as a stress-responsive gene linked to transcriptional adaptation, and IFNGR1 as a receptor component mediating type II IFN signaling. However, the available evidence does not uniformly establish these molecules as direct oncogenic drivers in EC. Therefore, their roles should be interpreted as potentially oncogenic, resistance-associated, or immune-modulatory depending on the experimental and clinical context. Although the IFN pathway has been associated with tumor suppression, chronic activation and associated stress from therapies can invoke certain IFN pathway components with oncogenic potential (Alspach et al., 2019). Recent studies in EC show dysregulated IFN pathway signaling may contribute to immune system exhaustion, adaptive resistance, and poor response to NAT (Wu et al., 2026). Within these components, IER2 and IFNGR1 are of interest for their role in immune modulation and resistance to therapy (Chen et al., 2023).

### 5.1 IER2: molecular functions and clinical relevance

As a stress-responsive gene, IER2 relays stress signals, including those from cytokines, growth factors, and DNA damage and interferons (Ceccaldi and Cejka, 2025; Tubbs and Nussenzweig, 2017). IER2 is an example of a transcription factor that generates an extracellular signal into an intracellular pathway that controls cell fate, mobility, and the regulation of the immune system (Kyjacova et al., 2021). Available evidence suggests that elevated or sustained IER2 expression may be associated with immune escape, checkpoint-related signaling, and reduced effector immune activity; however, direct causal evidence in EC remains limited (Chen et al., 2023). In terms of IER2’s functions, it is mostly associated with the aggravation of chronic, high-level, uninterrupted IFN signal transduction and the modification of ISG networks, which is characteristic of immune system exhaustion (Kyjacova et al., 2021). IER2 expression has been associated with treatment-resistance phenotypes in preclinical models and transcriptomic analyses; however, EC-specific studies directly linking IER2 to NAT response outcomes are currently limited (Kyjacova et al., 2021). The proposed mechanism—whereby IER2 activates stress-induced transcriptional programs that enable tumor cells to survive DNA-damaging treatment and sustain an immunosuppressive microenvironment—remains hypothesis-generating in the EC context and requires prospective validation.

### 5.2 IFNGR1: signaling dysregulation in radiotherapy and chemotherapy

The IFNGR1 is a ligand-binding unit of the IFN-γ receptor complex, and also acts as a primary controller of Type II IFN signaling (Chen et al., 2023). Normally, the activation of IFNGR1 occurs positively, driving antigen presentation, which results in the activation of macrophages, as well as the enhancement of cytotoxic immune responses (Tan et al., 2026). The situation changes when there is a continued exposure to IFN or when therapeutic stress is present. In these cases, the IFNGR1 signaling pathway becomes dysregulated, moving towards immune exhaustion and tumor adaptation. The procedure of radiotherapy and chemotherapy activates what is called robust IFN-γ signaling (Alspach et al., 2019). This is because immune cells and the DNA damage inflammatory response are both triggered (Kwon and Bakhoum, 2020). Sustained activation of IFNGR1 within tumor and stroma cells will drive higher levels of downstream JAK-STAT signaling, which leads to prolonged production of a series of genes, termed ISGs, as well as ICIs such as PD-L1 (Yi et al., 2021; Gao and Sun, 2026). This persistent signaling creates newer feedback loops that act in the suppression of T-cell activity and increase immune tolerance. Tumors with sustained IFNGR1–JAK–STAT activity may acquire transcriptional programs that support DNA-damage tolerance, immune evasion, and survival after cytotoxic therapy; however, this mechanism should be considered hypothesis-generating unless supported by direct EC-specific experimental evidence (Kwon and Bakhoum, 2020). This also leads to a lack of immune system detection and removal, also termed immune-mediated clearance. In EC, sustained IFNGR1-related signaling may be associated with reduced NAT responsiveness, though this relationship is largely inferred from mechanistic studies in other tumor types and from indirect evidence in EC transcriptomic datasets; prospective validation as a predictive biomarker for NAT response is still needed (Chen et al., 2023). Recently, it has been found that TMEM199 is a key modulator of IFN-γ-determined PD-L1 transcription. It binds CCDC115 and interacts with IFNGR1/2. This complex facilitates the trafficking to recycling endosomes for increased IFNGR1/2 recycling and upregulation of PD-L1. It has been suggested that TMEM199 is a therapeutic target for immunotherapy (Li et al., 2025).

### 5.3 Prognostic and predictive significance of IFN-Pathway components IER2 and IFNGR1

The potential of IFN pathway components to be oncogenically repurposed is of significant prognostic and predictive value to the clinical management of EC (Gao and Sun, 2026). Genes such as IER2 and IFNGR1 showing elevated expression have been associated in some analyses with immunosuppressive TME features and immune exhaustion marker profiles, though prospective prognostic validation in EC is still needed (Han et al., 2025; Li et al., 2025). These genes qualify as potential prognostic biomarkers for the determination of patients at high risk of developing resistance to NAT and recurring disease. Even more predictive, IFN pathway components hold value for patient stratification and therapeutic tailoring (Chen et al., 2023). Patients with IFN-induced exhaustion signatures may achieve very little benefit from standard NAT alone. They may respond better to combination therapies such as ICB or IFN pathway modulation (Topalian et al., 2023). In addition, changes in gene expression of IFN-related genes during therapy may serve to predict the therapeutic effect or failure of the therapy. Overall, these observations provide evidence for the clinical importance of IFN pathway components for EC (Li et al., 2025). Addressed dysregulated IFN signaling and combinations of therapies may provide a means for overcoming immune exhaustion, enhancing the NAT, and improving the clinical outcomes (Chen B. et al., 2026).

## 6 Neoadjuvant treatment strategies in EC

For advanced EC, NAT is increasingly becoming a fixture in management due to its ability to downstage tumors, improve surgical resectability, and eliminate micrometastatic disease (Wu et al., 2026). Nevertheless, the therapeutic benefit appears to be somewhat stochastic and heterogeneous and is likely due to the interactions among tumor biology, immune modulation, and stress response. Studies continue to indicate that neoadjuvant combinations of therapies carry effects which go beyond direct ‘targeted’ necrosis of tumor cells and include robust alterations of the TIME at least in cases where NATs activate IFN signaling (Liu et al., 2025).

### 6.1 Chemotherapy-based neoadjuvant regimens

**FIGURE 5:**
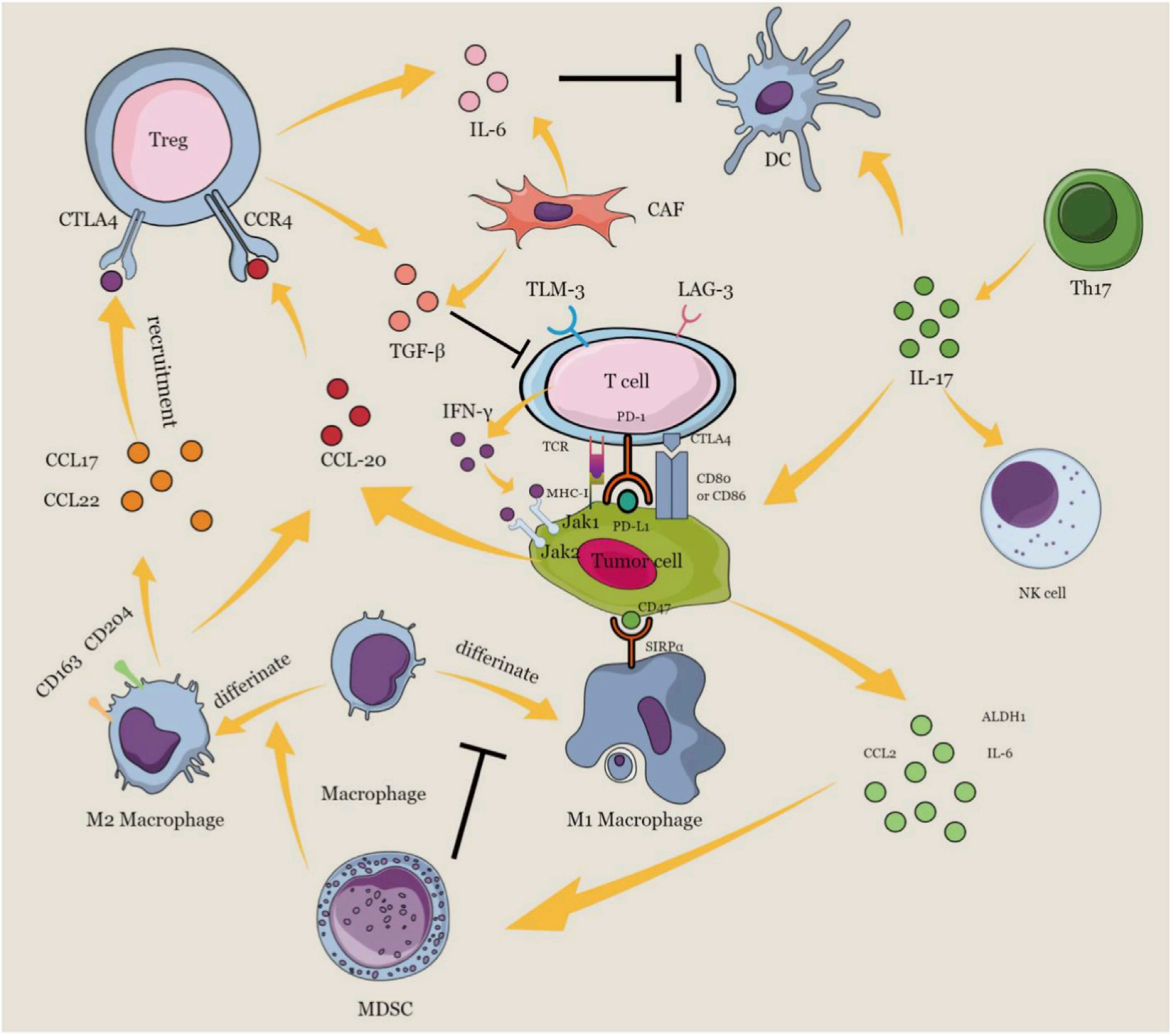
The mechanism of immune resistance in EC (Fang et al., 2022). CTLA-4, CCR4, CCL2, CCL17, CCL20, CCL22 represent cytotoxic T lymphocyte-associated proteins, TIM-3, T cell immunoglobulin and mucin-domain containing-3; LAG-3, lymphocyte-activation gene 3; CAF, cancer-associated fibroblast; JK, Janus kinase. Reprinted from Fang P, Zhou J, Liang Z, Yang Y, Luan S, Xiao X, Li X, Zhang H, Shang Q, Zeng X and Yuan Y (2022) Immunotherapy resistance in esophageal cancer: Possible mechanisms and clinical implications. Front. Immunol. 13:975,986. Doi: 10.3389/fimmu.2022.975986.

\Standard neoadjuvant chemotherapy regimens differ between subtypes: in EAC, FLOT (docetaxel, oxaliplatin, leucovorin, fluorouracil) and ECF/ECX-based regimens are commonly used, whereas in ESCC, platinum-based regimens combined with taxanes or fluoropyrimidines remain standard (Lopez et al., 2020; Zhao et al., 2022). The immunological consequences of these regimens, including IFN pathway activation, may therefore differ between subtypes. The goal of these regimens is to achieve a reduction in tumor burden and, subsequently, to sensitize the tumors for surgical resection. In addition to tumor cell death and the potential promotion of the more immunogenic variant of cell death with the release of tumor-associated antigens and danger-associated molecular patterns (DAMPs), chemotherapy may stimulate further immune activity (Chen et al., 2025). The immune activity, however, may be countered by the effects of repeated or prolonged exposure to chemotherapy, which may induce an unresponsive or immunosuppressive state. In chemotherapy, the inflammation and interferon signaling can lead to immune exhaustion in a sustained or chronic manner (McLaughlin et al., 2020). The upregulation of ISGs and ICIs can result from post-chemotherapy responses and may further limit the durable anti-tumor immunity due to the treatment (Gao and Sun, 2026). This means that, while the initial impacts of neoadjuvant chemotherapy may boost immune recognition, they may also cause the TME to be more prepared for adaptive immune resistance (Wu et al., 2026). The mechanistic details of immune resistance in EC are given in Figure 5 (Fang et al., 2022).

### 6.2 Radiotherapy and chemoradiotherapy approaches

In the neoadjuvant setting, radiotherapy, with or without chemotherapy, is one of the cornerstones of treatment (Miller et al., 2026). Cell death, or apoptosis, from radiation-induced DNA damage, activates immune pathways, including the cGAS-STING pathway, which is accountable for type I interferon synthesis (Li et al., 2026; Hopfner and Hornung, 2020). Therefore, the combination of radiotherapy with other forms of immunotherapy is rational (Pointer et al., 2022; Galluzzi et al., 2023). In numerous clinical studies, chemoradiotherapy (CRT) has resulted in better local control and improved rates of pathological responses than chemotherapy alone (Chen B. et al., 2026). However, the immunological effects of CRT are multifaceted and dependent upon numerous variables. While acute, post-radiation, type I-interferon signaling can improve the exhibition of tumor antigens and the priming of T cells, it can cause T-cell and potentially other immune cell exhaustion (Arimoto et al., 2018). It upregulates the immunological synapse and ultimately, tumor immune evasion (Li et al., 2023). These mechanisms may provide some explanation as to why better pathological responses are not always associated with improved long-term outcomes (Zhang B. et al., 2023). An interrelationship between TME and neoadjuvant chemoradiotherapy has been reviewed in EC patients. The analysis has observed a high expression of PD-L1 after the chemoradiotherapy, which was linked with poor pathological response. It was illustrated that CD8, CD4, CD3, and PD-L1 were potential markers in envisaging pathological response (Wang et al., 2024).

### 6.3 Sequencing and integration of radiotherapy and chemotherapy: Biological implications

**FIGURE 6:**
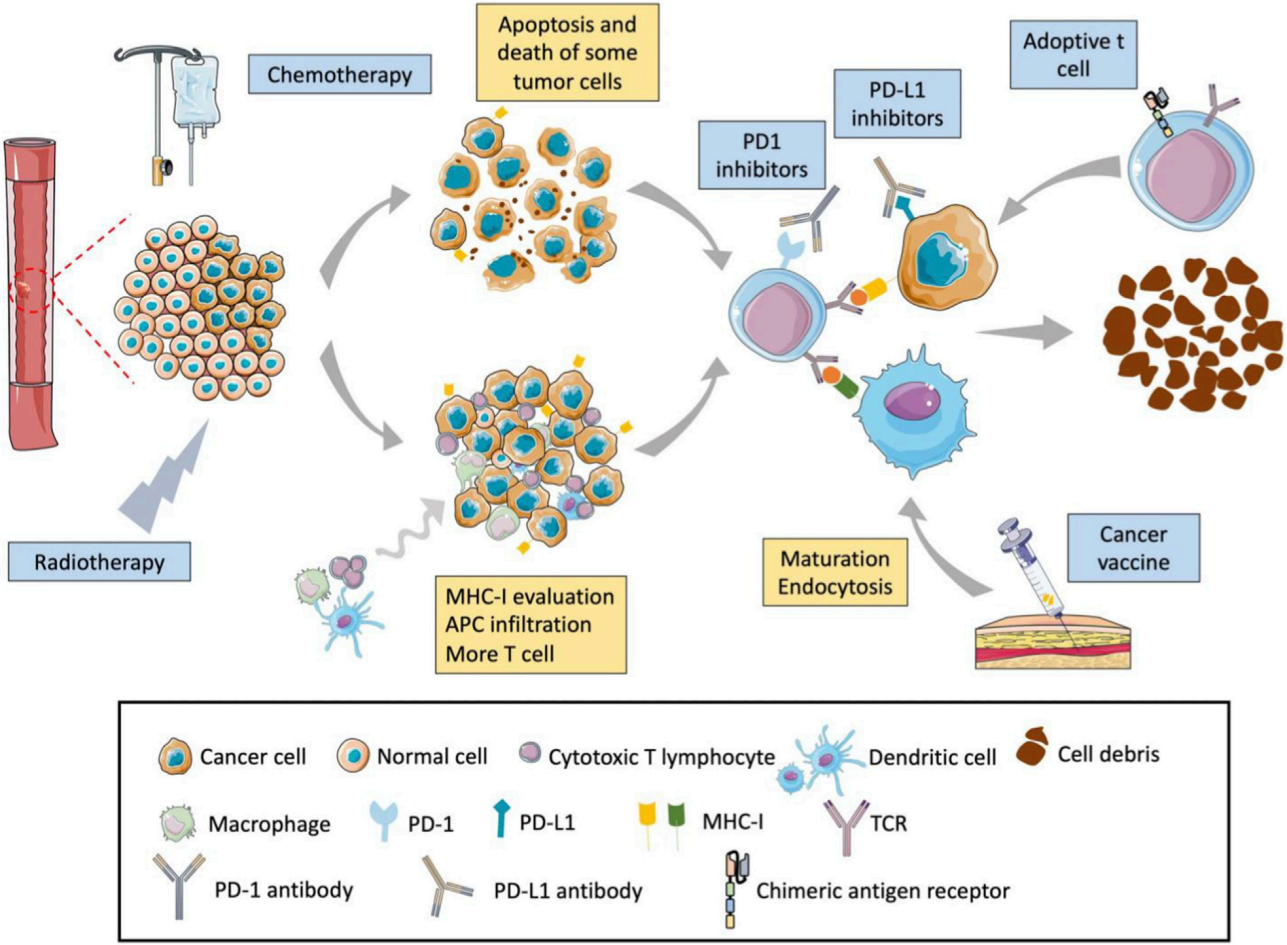
Mechanism of radiotherapy-chemotherapy combination in esophageal cancer (Wang H. et al., 2022). Radiotherapy kills cancer cells, which release tumor-specific antigens to help in finding more cancer cells. Cellular DNA is destroyed, producing neoantigens to trigger an immune response. It upregulates MHC-I expression in tumor cells with the modulation of the TME. The migration of cytotoxic T cells and upregulation of the PD-L1 enhances the therapeutic effect of PD-L1 antibodies. Reprinted from Wang H, Xu Y, Zuo F, Liu J and Yang J (2022) Immune-based combination therapy for esophageal cancer. Front. Immunol. 13:1,020,290. Doi: 10.3389/fimmu.2022.1020290.

Because the terms radiochemotherapy and chemoradiotherapy are often used interchangeably, this section focuses specifically on whether the timing, sequencing, and intensity of radiation and chemotherapy may differentially affect IFN activation, DNA damage response, and immune exhaustion. The order of administration of each modality, that is, chemotherapy preceding or following radiotherapy, radiochemotherapy vs. chemoradiotherapy, has significant implications in the biological and immunological responses (Zhang B. et al., 2023). In chemoradiotherapy, where chemotherapy is given at the same time as radiotherapy, it can lead to increased radiosensitivity and a further enhancement of the IFN-related immune response (Miller et al., 2026). Contrarily, immune stress may be worse in this scenario and can lead to immune-mediated exhaustion (IME) sooner in susceptible tumors. In radiochemotherapy, where radiotherapy is given before or after chemotherapy, immune responses can be modulated differently because of the temporal separation of immune activation and genotoxic stress (Miller et al., 2026; Pointer et al., 2022). Several recent studies indicate that the sequencing of treatments can impact the IFN pathways, specific immune checkpoint pathways (ICPs), and the duality of immune activation and exhaustion. Such tumors with a dysregulated IFN pathway, constitutively or in the oncogenically activated state, will have a varied response to the regimen, suggesting that such approaches should be taken in a biomarker-directed manner (Chen et al., 2023). The biological effects of NATs in EC are significant and go far beyond the goal of tumor debulking (Wu et al., 2026). In extreme cases, modulations of immune exhaustion and IFN pathways are the most important factors in determining response or resistance to treatment (Wan et al., 2026). It thereby underscores the need for a focus in order to fully harness the beneficial aspects of NATs (Figure 6 **)**. A rational approach must be made to the combinations of treatments to protect and prolong anti-tumor immunity, and to reduce or reverse immune exhaustion (Malekzada et al., 2025).

## 7 Neoadjuvant therapy and immune exhaustion

The beginning of the negative immune impact from NAT can start before the immune alteration from the reduction in the size of the tumor from the therapy (Pointer et al., 2022). Chemotherapy and radiotherapy affect the TIME in different ways (Miller et al., 2026). TIME can change the immune activation, immune suppression, and immune exhaustion (Liu et al., 2025). Some immune activation can indeed lead to some beneficial effects for the therapy and aid in the elimination of the tumor. However, the dominant immunological consequence of prolonged NAT is immune dysfunction, which represents a critical barrier to durable treatment responses in EC (Zhang B. et al., 2023).

### 7.1 Therapy-induced immune modulation

The first and the most obvious positive immune change caused by neoadjuvant chemotherapy and radiotherapy is the immune activation in the form of a cell ‘kill’ response from the tumor (Zhang B. et al., 2023). These responses bring about the creation and the release into the microenvironment of tumor antigens (Miller et al., 2026). Caveolin-1 (CAV1) has exhibited its influence on immune cell infiltration in TME and has a promising response towards immunotherapy in EC. The expression of CAV1 is downregulated in EC and is positively linked with immunomodulation, cancer immunity and immune checkpoints. It has been claimed that CAV1 may be employed in the ICIs’ response towards clinical therapies (Zhang R. et al., 2023). A TME-reelated prognostic gene AIF1, has influenced immune infiltration and TIGIT expression and has emerged as a promising predictor of prognosis in EC (Xu et al., 2022). The dysfunction of a protein DPY-30 homolog (DPY30), a key member of histone H3K4 methylation catalase, has been linked with a positive interaction against various immune cell infiltrations. It has been suggested as a prognostic and therapeutic target against ESCA (Mei et al., 2022). An N6-methyladenosine modification in an RNA molecule has been linked with poor prognosis and immune cell infiltration in EC (Sheng et al., 2023). Other molecules that also contribute to the immune response are DAMPs and several inflammatory signaling molecules. The net result of this is that the immune response can lead to a greater response from the system to the antigen. It subsequently can lead to the recruitment of immune effector cells and even a positive response and an increase in the response from the cytotoxic T cells. In a relationship where some of these positive immune responses were seen, there becomes a cellular-level tumor regression and may end up in an improved clinical response (Chen B. et al., 2026). Unfortunately, these positive impacts and immune responses are often observed and can start to plummet due to the prolonged and continued success of the immune response. The cytotoxic cells begin to undergo a crisis to a level of suppression, including for the Treg cells and the MDSCs (Cui et al., 2025). On the other hand, continual stimulations lead to limiting the early immune responses, which will result in the development of chronic inflammatory responses and dysregulated signaling cascades. It will ultimately permit the tumor to elude (McLaughlin et al., 2020).

### 7.2 IFN pathway activation following radiotherapy and chemotherapy

NAT and its resulting immune activation prominently feature the IFN signaling pathway (Hopfner and Hornung, 2020). Radiation-induced DNA damage activates the cGAS-STING innate immune sensing pathway, triggering type I IFN production—a mechanism documented in EC *in vitro* models and corroborated by clinical tumor sample analyses (Li et al., 2026). Similarly, while chemotherapy activates IFN signaling as a consequence of the stress DNA damage and the associated inflammatory response, both pathways act to improve antigen presentation and promote T cell priming early on in treatment (Kwon and Bakhoum, 2020). Notwithstanding the positive benefits, IFN pathway activation in its persistent state can result in negative outcomes (Pang et al., 2025). Continuous states of IFN signaling promote the expression of ICIs such as PD-L1 and increase the inhibitory signaling within the TME (Lin et al., 2024; Sun et al., 2018; Battaglia et al., 2023). Persistent ISG activation has been associated with T-cell dysfunction across multiple cancer types (Gao and Sun, 2026). In the case of EC, increased IFN pathway activity following the application of NAT is correlated with treatment resistance and poor results (Wan et al., 2026).

### 7.3 Molecular mechanisms linking neoadjuvant response to immune exhaustion

Several interrelated molecular processes govern the change from immune activation to immune exhaustion after NATs (Wu et al., 2026). Generally, the immune system reacts to NATs as though there was an injury through the release of IFN signaling which subsequently leads to the formation of what are termed ‘immune checkpoints’ that suppress T cell activation and allow tumor cells to survive (Malekzada et al., 2025). In addition, the inhibitory IFN signaling pathway is also involved in the DNA damage response (DDR) pathways, which can lead to adaptation to collateral damage while the TME remains suppressive (Xia et al., 2026). Moreover, signaling components of the oncogenic IFN pathways, such as IER2 and IFNGR1, exacerbate the situation through the activation of chronic IFN signaling loops. These processes can upregulate immune checkpoints, inhibit effector immune function, and promote immune resistance to radiation and chemotherapies. Consequently, NAT and resultant immune exhaustion contribute to the failure of cancer therapies (Wu et al., 2026). These findings highlight the paradoxical immune consequences of NAT in esophageal cancer: while these therapies can initiate an antitumor immune response and promote tumor shrinkage, excessive or sustained IFN signaling may lead to immune exhaustion and limit the durability of treatment response. Consequently, while these are effective at inciting an immune response and tumor shrinkage, excessive IFN signaling leads to immune exhaustion and a lack of long-lasting responses. Interventions that reverse these aberrant immune pathways enhance the effectiveness of NATs and improve patient survival (Malekzada et al., 2025).

## 8 Therapeutic resistance and mechanisms of immune escape

The main barriers to achieving clinically meaningful benefits in the treatment of cancer, specifically, NAT, remain therapeutic resistance and immune evasion (Malekzada et al., 2025). The primary cause of both of these characteristics is driven by the intrinsic genetic changes of a tumor. However, there is significant evidence to support that the changes made by therapy-induced immune remodeling also play a key role. Immune exhaustion and chronic signaling by the immune IFN are adaptive mechanisms used by the tumor to resist therapeutic stress and avoid destruction by the immune response (Li et al., 2023).

### 8.1 IFN-mediated adaptive resistance

Adaptive resistance phenomenon entails a response to therapy-induced changes that allow for the survival of tumor cells under selective pressure (Wan et al., 2026). IFN signaling is a conduit for this mechanism, and acute IFN activation is essential for the improvement of antitumor immunity. However, continuous exposure to IFN leads to the transcription of programs that improve the survival of tumor cells, immune evasion, and resistance to cytotoxic therapies (Li et al., 2023). EC prolonged IFN signaling post-chemotherapy or radiotherapy exhibits phenomena such as the upregulation of immune checkpoint ligands, changes to the antigen-presenting machinery, and the activation of survival pathways (Miller et al., 2026; Zhang B. et al., 2023). These phenomena blunt immune effector functions and decrease the likelihood of a response to further treatment. Tumor cells manipulate IFN-stress pathways to improve tolerance towards DNA damage, evasion of apoptosis, and remain in a dormant or therapy-resistant state (Xia et al., 2026; Kwon and Bakhoum, 2020). Most adaptive resistance mechanisms are reversible, which lends to the perspective that strategic changes to IFN signaling and the downstream pathways would improve the disease prognosis.

### 8.2 Exhaustion markers and treatment refractoriness

Immune exhaustion occurs when there are consistent signs of inhibitory receptors, and the functionality of effector immune cells is obstructed. In EC, the increased signs of immune exhaustion, such as PD-1, PD-L1, CTLA-4, TIM-3, and LAG-3, have been correlated with poor responses to NAT and greater chances of recurrence (Lin et al., 2024; Cui et al., 2025; Yang et al., 2022; Yi et al., 2021). Treatment refractoriness is often associated with exhaustion signatures on the transcriptional level that are associated with long-term chronic antigen exposure and sustained IFN signaling. Exhausted T cells progressively lose effector function, including cytokine production, proliferative capacity, and cytotoxic activity. They are thus unable to destroy remaining tumor cells after NAT (Malekzada et al., 2025). At the same time, the expansion of immune suppressive cells in the TME continues to reinforce immune tolerance and resistance. The presence of exhaustion signatures before and during the course of treatment could be indicative of poor therapeutic outcomes. Furthermore, this reinforces the rationale for targeting such markers in combination approaches to reinvigorate antitumor immunity to overcome resistance (Wan et al., 2026).

### 8.3 Tumor evolution under neoadjuvant pressure

NAT imposes selective pressure on tumor-cell populations, potentially leading to clonal evolution and immune adaptation (Wu et al., 2026). The tumor cells that can withstand the initial rounds of treatment possess certain features on the TME that are linked to treatment resistance and even transform the microenvironment to mitigate the NATs (Nishida and Andoh, 2025). These features include the upregulation of persistent stress response and immune evasion signaling pathways, alteration of the IFN pathway, and even modification of the TME to promote immune evasion (Yi et al., 2021). Therefore, in the presence of sustained neoadjuvant pressure, tumors may evolve to manifest phenotypes that are typically associated with immune subversion. These include attenuated immunogenicity, deficient antigen processing and presentation, and increased immune cell repression (Li et al., 2023). IFN signaling contributes to the immune subversion in a selective manner that promotes the retention of tumor cell phenotypes that are able to survive in the poor immune environment caused by immune depletion (Malekzada et al., 2025). The process of dynamic evolution exemplifies the shortfalls that come with singular treatment approaches and places emphasis on the importance and utility of adaptive treatment approaches determined by certain tumor biomarkers. The ability to shape tumor evolution and immune escape during the course of NATs is central to designing resistance-evading interventions and achieving sustained tumor control (Wan et al., 2026).

## 9 Integrated approaches to immune exhaustion

Understanding immune exhaustion is crucial to overcoming barriers to improving the effectiveness of neoadjuvant therapy in EC (Malekzada et al., 2025). Current knowledge of the IFN pathway dysregulation, checkpoint-mediated immune suppression, and therapy-induced adaptive resistance are the building blocks of rational combination strategies (Wan et al., 2026). These strategies aim to restore and reinvigorate antitumor immune responses while preserving cytotoxic capacity. Addressing these mechanisms could improve therapeutic response, along with a sustained clinical benefit (Zhou et al., 2022).

### 9.1 Targeting IFN pathway oncogenes

Therapeutic modulation of these pathways may restore immune competence and increase tumor susceptibility to NAT, but this strategy requires further preclinical and clinical validation. Some preclinical models suggest reduced checkpoint expression, partial restoration of T-cell function, and improved cytotoxic activity, supporting further investigation of these strategies in carefully designed clinical studies (Chen B. et al., 2026; Akumaga et al., 2025; Wang et al., 2018; Fang et al., 2022; Butterfield and Najjar, 2024).

### 9.2 Integration of immunotherapy with neoadjuvant regimens

**FIGURE 7:**
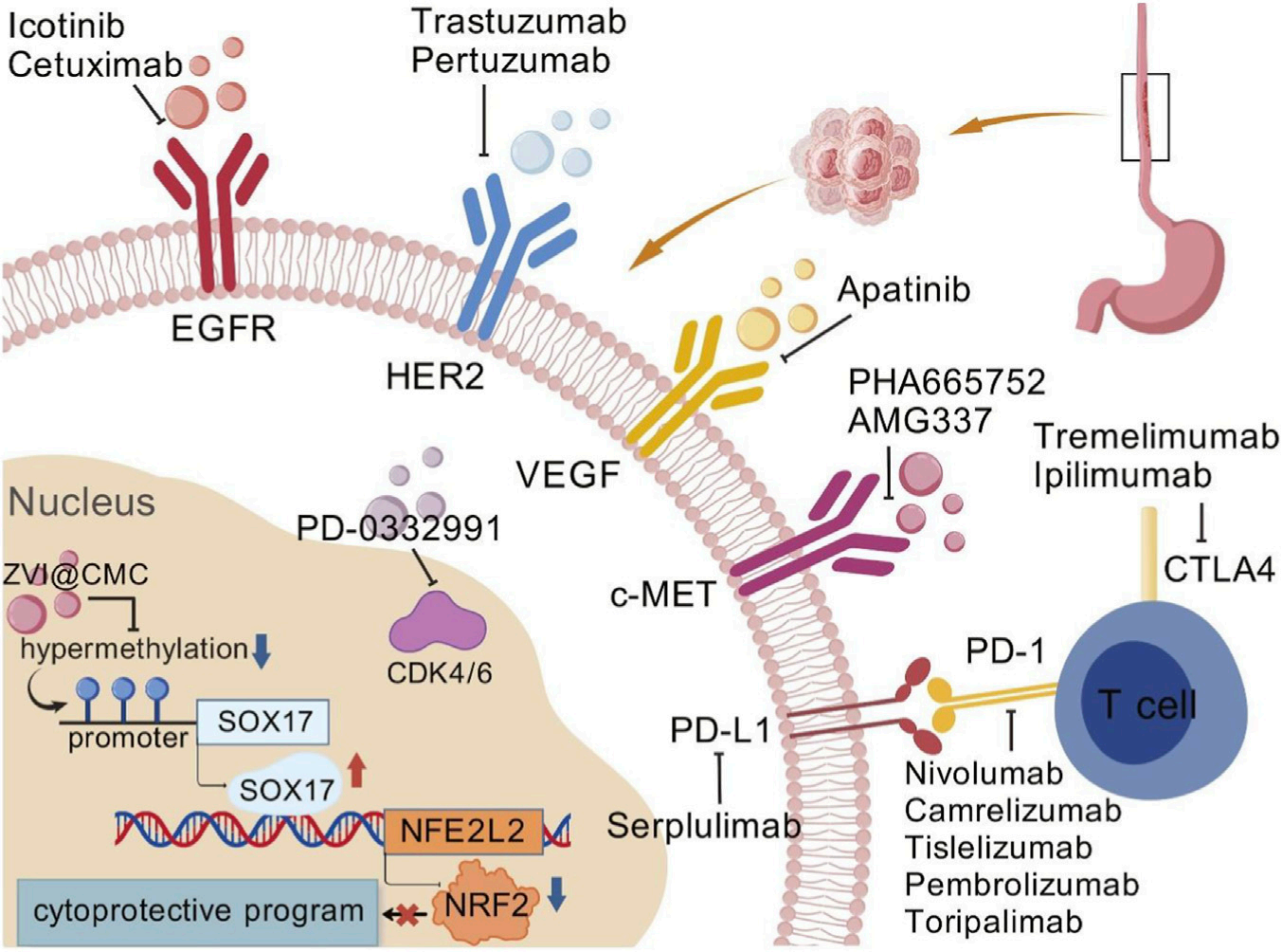
Potential drug targets of esophageal cancer (Pei et al., 2025). Reprinted from G. Pei, Z. Liang, B. Gu, L. Shi, Z.-X. Liu, S. Gao, Interdiscip. Med. 2025, 3, e20240,105. https://doi.org/10.1002/INMD.20240105.

The addition of immunotherapy to standard NATs is an opportunity to prevent the immune exhaustion that is the result of these therapies (Pointer et al., 2022; Galluzzi et al., 2023). Targeting PD-1/PD-L1 and CTLA-4, through ICB, serves to strengthen exhausted T cells and improve both cytotoxic activity and overall antitumor immune response (Lin et al., 2024; Yang et al., 2022; Yi et al., 2021; Topalian et al., 2023). Furthermore, state-of-the-art STING agonists, oncolytic viruses, and some cytokine therapies, used in conjunction with chemotherapies and radiotherapies, may enhance the immune response through IFN pathways while alleviating exhaustion (Li et al., 2026). In the neoadjuvant setting, the optimum effect of most immunotherapies is dependent on factors such as the timing, sequencing, and quantity of each therapy employed (Wu et al., 2026). Because T cell priming may be positively impacted by the early addition of these therapies, and because the length of treatment may result in an increase in the level of exhaustion, these factors must be closely controlled. Innovative combination approaches that are based on solid and rational mechanistic understanding of IFN signaling and immune exhaustion are critical (Malekzada et al., 2025). However, several potential risks of combining IFN-pathway modulation with NAT must be acknowledged. Broad upstream IFN suppression (e.g., via JAK inhibition) risks impairing the beneficial acute antitumor IFN response, potentially reducing treatment efficacy. Combination timing is critical: immunotherapy added before sufficient NAT-induced antigen release may be ineffective, while delayed addition after irreversible exhaustion is established may equally fail. Furthermore, targeting downstream exhaustion pathways (PD-1/PD-L1) may not fully reverse multi-receptor exhaustion phenotypes. Finally, ESCC and EAC may respond differently to combination strategies given their distinct immune profiles, underscoring the need for subtype-stratified trial designs. Some of the potential targets are displayed in Figure 7.

### 9.3 Biomarker-driven patient stratification

Given the heterogeneity of IFN pathway activation and immune exhaustion across EC patients, biomarker-driven patient stratification represents a promising approach to guiding treatment selection. Several candidate biomarkers have been proposed, though none has yet been prospectively validated for routine clinical use. IER2 and IFNGR1 expression can be assessed by immunohistochemistry (IHC) on formalin-fixed paraffin-embedded (FFPE) tumor tissue, or quantified at the RNA level using reverse-transcription PCR or next-generation sequencing panels. IFN-stimulated gene (ISG) signatures are best captured through multiplex gene expression profiling platforms such as NanoString or bulk RNA sequencing, which allow simultaneous quantification of multiple ISG transcripts. Inhibitory checkpoint markers, including PD-1, PD-L1, TIM-3, and LAG-3, are most commonly evaluated by IHC; multiplex immunofluorescence enables co-localization of these markers with specific immune cell subsets (e.g., CD8^+^ T cells), providing a more functionally informative readout (Lin et al., 2024; Cui et al., 2025; Yang et al., 2022). Dynamic changes to these biomarkers during NAT may further refine treatment in real-time, allowing for adjustments to maximize immune reactivation and therapeutic response. To integrate biomarker-driven stratification with IFN-targeted and immunomodulatory strategies is to move toward personalized NAT (Malekzada et al., 2025). Such approaches seek to do more than improve pathological responses, but also to prevent immune evasion and to provide long-lasting disease control in EC (Li et al., 2023).

Several important limitations affect the clinical applicability of these biomarkers. First, intratumoral spatial heterogeneity means that a single-core biopsy may not be representative of the overall tumor immune landscape. Second, the optimal sampling time point remains undefined: pre-treatment baseline biopsies are most practical for prediction, whereas post-NAT residual tumor tissue may better capture therapy-induced immune remodeling but is available only after treatment decisions have been made. Third, none of the proposed IFN-related or exhaustion biomarkers currently has a validated expression threshold that reliably separates responders from non-responders. Fourth, differences between ESCC and EAC in immune contexture may mean that biomarker thresholds identified in one subtype do not translate to the other. Dynamic changes to these biomarkers during NAT may ultimately help refine real-time treatment adjustments, but this requires prospective study designs with serial tumor sampling or validated liquid biopsy approaches.

## 10 Clinical implications and translational perspectives

Understanding the mechanisms of immune exhaustion and dysregulation of the IFN pathway in EC offers myriad clinical possibilities (Chen B. et al., 2026). It has been displayed that metformin, an antidiabetic agent, is involved in the reprogramming of TME in ESCC in a phase II trial. Metformin improved phagocytosis of ESCC cells during *in vitro* studies. In the ESCC mouse model and in humans, it initiated AMPK activation and STAT3 inactivation with the modifications in the synthesis of cytokines like TNF-α, TNF-γ and IL-10 in immune cells (Wang et al., 2020). In a single-arm phase two trial, chemoimmunotherapy followed by chemoradiotherapy and immunotherapy illustrated potential benefits with good survival outcomes with substantial safety profiles, thereby offering a better alternative treatment for patients with advanced ESCC (Chen H. et al., 2026). Applying various approaches for the betterment of patient health opens avenues for the improvement of the outcomes of NAT (Malekzada et al., 2025). It offers support for certain therapeutic decisions and leads to the development of personalized treatment approaches based on the molecular and immune profiles of the patient (Table 1).

**TABLE 1:** A summary of the clinical trials of esophageal cancer (Pei et al., 2025).

Therapy	Therapeutic target	Drugs	Trial registration number	Clinical phase
Target therapy	EGFR	Icotinib	NCT01855854	II
	EGFR	Cetuximab	NCT00096031	II
	HER2	Trastuzumab + Deruxtecan	NCT02564900	I
	HER2	Trastuzumab + Deruxtecan	NCT03329690	II
	HER2	Trastuzumab + Deruxtecan	NCT04014075	II
	VEGF	Bevacizumab	NCT00450203	II - III
Immunotherapy	PD-1	Nivolumab	NCT03143153	III
	PD-1	Nivolumab	NCT02569242	III
	PD-1	Serplulimab	NCT03958890	III
	PD-1	Camrelizumab	NCT03691090	III
	PD-1	Toripalimab	NCT03829969	III
	PD-1/PD-L1 + CTLA4	Durvalumab + Tremelimuma	NCT03377400	II
	PD-1/PD-L1 + CTLA4	Nivolumab + Ipilimumab	NCT01928394	II
	PD-1	Nivolumab	NCT02872116	III
	PD-1 + CTLA4	Nivolumab + Ipilimumab	NCT02872116	III
Target therapy + immunotherapy	PD-1 + HER-2	Pembrolizumab + Trastuzumab	NCT02954536	II
	PD-1 + HER-2	Pembrolizumab + Trastuzumab	NCT03615326	III
	PD-1 + HER-2	Margetuximab + Pembrolizumab	NCT02689284	Ib-2
	PD-1 + VEGFR2	Camrelizumab + Apatinib	NCT03736863	II
	PD-1 + VEGFR2	Ramucirumab + Pembrolizumab	NCT02443324	Ia/b
	PD-1 + EGFR	Serplulimab + HLX07	NCT05221658	II

EC, esophageal cancer; ESCC, esophageal squamous cell carcinoma; EAC, esophageal adenocarcinoma; NAT, neoadjuvant therapy; ICI, immune checkpoint inhibitor; IFN, interferon.

### 10.1 Predicting NAT response

The ability to accurately predict the response to NAT in EC is an ongoing clinical dilemma (Wu et al., 2026). Tumor-related attributes such as tumor stage, the specific histology, any available imaging-based histology and structural/functional biomarker analysis provide an incomplete picture of the scope of therapeutic failure mitigation. The development of predictive models incorporating immune and molecular biomarkers, especially those focused on the activity of the IFN pathway and state of immune exhaustion, is more likely to yield improved estimates of therapeutic success. Profiles such as IER2 and IFNGR1, immune exhaustion profiles such as PD-1, TIM-3, LAG-3, and gene expression profiles of IFN are likely to be present in patients who are at the highest risk of potential treatment failure (Cui et al., 2025; Yang et al., 2022). Monitoring for sustained IFN signaling or exhaustion markers during the course of NAT may help identify patients who would benefit from treatment escalation or the addition of immunotherapy, though the optimal timing and modality of such monitoring remain to be defined in prospective studies (Song et al., 2026). Ultimately, these biomarker-driven strategies are aimed at decreasing the indices of treatment to recurrence, improving the associated pathological response rates, and improving the overall survival time of patients (Chen B. et al., 2026).

### 10.2 Implications for personalized treatment

The understanding of the iterative nature of immune exhaustion and the biphasic role of IFN signaling is pivotal for the customization of treatment plans (Chen B. et al., 2026). Neoadjuvant chemotherapy or radiotherapy combined with immune modulators, such as PD-1/PD-L1 inhibitors or JAK-STAT pathway inhibitors, and potentially new therapies targeting the IFN pathway, may be warranted for patients with a specific tumor type with sluggish immune activity and with immune axis exhaustion (Miller et al., 2026; Lin et al., 2024; Cui et al., 2025; Yang et al., 2022; Yi et al., 2021). In contrast, some patients with no or little immune exhaustion and no dysregulation of the IFN pathway may only need traditional NATs. It would spare the patient the potential side effects of more than one immunotherapy (Galluzzi et al., 2023). In real-time, during treatment, it may be possible to personalize the treatment approach based on the results of the biomarkers for IFN and immune exhaustion (Malekzada et al., 2025). The more closely proximate the biomarker’s signals to the tumor-immune response, the more real-time the treatment will be. The more closely integrated the molecular, immune, and clinical data into treatment algorithms, the more advanced the practice of precision oncology for EC will be. Understanding these mechanisms will greatly aid clinicians and improve the efficacy of overcoming immune resistance and the prognosis of patients (Wan et al., 2026) (Table 2).

**TABLE 2:** Evidence level of IFN-related therapeutic and biomarker strategies in EC.

Strategy	Proposed mechanism	Evidence level	EC-specific evidence	Clinical readiness
IER2 modulation	May reduce stress-adaptive immune suppression	Preclinical/hypothesis-generating	Limited	Not clinically validated
IFNGR1/JAK–STAT modulation	May reduce chronic IFN-driven checkpoint upregulation	Preclinical/translational	Limited to moderate	Requires prospective validation
PD-1/PD-L1 blockade with NAT	May reverse immune exhaustion	Clinical and translational	Moderate to strong depending on setting	Under active clinical evaluation
TIM-3/LAG-3 targeting	May reverse deeper exhaustion phenotypes	Early clinical/preclinical	Limited	Investigational
IFN-related gene signatures	May predict NAT response or immune exhaustion	Retrospective/exploratory	Limited	Requires validated cutoffs

## 11 Future directions and emerging therapeutic opportunities

Further comprehension of immune exhaustion, IFN signaling, and response to NAT in EC has facilitated novel pathways for research and clinical application (Malekzada et al., 2025). Future strategies focus on circumventing the limitations attributed to conventional methods in order to translate mechanistic understanding into sustained therapeutic benefits (Chen B. et al., 2026).

### 11.1 Focus on the dysregulation of the IFN pathway

Dysregulated IFN signaling is both an obstacle and a potential clinical target. Restoring immune dysfunction without allowing the tumor to adapt can be achieved through innovative means focused on the selective modulation of IER2 and IFNGR1 (Li et al., 2025). Small molecule inhibitors, RNA interference, or monoclonal antibodies directed to specific targets can provide therapeutic strategies for the chronic state of IFN-induced immune exhaustion (Tseng et al., 2025; Su et al., 2024).

### 11.2 Rational combination therapies

The integration of immunotherapy with conventional neoadjuvant regimens is anticipated to substantially transform the pathological responses and survival outcomes (Pointer et al., 2022; Malekzada et al., 2025). The combination of ICB, STING pathway agonists or cytokine modulators with chemotherapy or radiotherapy is expected to work in the context of short-term IFN-mediated immune activation and is also predicted to reduce the immune exhaustion (Miller et al., 2026; Topalian et al., 2023; Hopfner and Hornung, 2020). The most important issue in the field is still determining the proper order and timing of these different approaches (Zhang B. et al., 2023).

### 11.3 Molecular profiling biomarker-based precision oncology

Multi-parameter bio-markers such as IFN pathway activity, immune exhaustion, and tumor mutation profiles help define patient grouping and tailoring specific treatment plans (Wan et al., 2026). If patient responses to these markers are monitored in real-time throughout NAT, it may be possible to make adaptive changes that optimize treatment effectiveness while minimizing harmful side effects (Malekzada et al., 2025).

### 11.4 Capturing tumor-immune evolution

The study of tumor evolution under therapeutic pressure has great potential for predictive modeling and adaptive treatment (Wen et al., 2024). Computational and single-cell techniques can be used to analyze clonal diversity, IME, and IFN adaptation, which are instrumental in designing and implementing strategies to circumvent resistance (Wan et al., 2026).

### 11.5 Clinical trials and translational research

More early-phase clinical and preclinical studies are required to confirm the safety, efficacy, and mechanisms of action for strategies that are immune-modulatory and IFN-targeted (Hoeppner and Nimczewski, 2025). Rapid clinical uptake and improved patient outcomes of trial designs that include adaptive translational objectives, such as immune exhaustion reversal and modulation of the interferon pathway, will be promoted (Chen B. et al., 2026). These strategies represent a significant shift to more precise and mechanism-based approaches in EC (Malekzada et al., 2025). Targeting the interplay between NAT, immune exhaustion, and IFN signaling will help overcome therapeutic resistance and improve long-term survival in the patient population (Wan et al., 2026).

### 11.6 Limitations of the current evidence

Several limitations should be acknowledged. First, much of the current evidence linking IFN pathway dysregulation to immune exhaustion and treatment resistance is derived from preclinical studies, retrospective transcriptomic analyses, or studies in non-esophageal cancers. Second, EC includes biologically distinct subtypes, particularly ESCC and EAC, and many studies do not report subtype-specific immune or molecular findings. Third, NAT regimens vary substantially in chemotherapy agents, radiation dose, treatment sequence, and timing of tissue sampling, limiting direct comparison across studies. Fourth, IER2 and IFNGR1 remain incompletely validated as predictive biomarkers for NAT response in EC. Fifth, immune exhaustion is dynamic and spatially heterogeneous; therefore, single pretreatment biopsies may not fully capture therapy-induced immune remodeling. Finally, prospective studies integrating serial tissue sampling, spatial immune profiling, functional assays, and clinical outcomes are needed before IFN-pathway biomarkers can be incorporated into routine treatment selection.

## 12 Conclusion

EC as a malignancy continues to be a clinical challenge, as there is a poor and varying response to NAT, and patients continue to have poor long-term outcomes. More recently, studies have suggested immune exhaustion, partially due to the dysregulation of certain IFN signaling and IFN pathway components such as IER2 and IFNGR1, to be a key contributor to immune therapy resistance and progressive disease. Chronic IFN activation not only reinforces T-cell exhaustion and immune checkpoint overexpression but also promotes adaptive tumor responses, which further compromise the effectiveness of chemotherapeutic and radiotherapeutic interventions. Understanding these pathways provides a rationale for translational studies, but clinical implementation will require prospective validation. The use of dysregulated IFN pathway targeting, the combination of immunotherapy with NAT, and the use of biomarkers to improve patient selection are potential therapeutic strategies to improve the immune response and clinical outcomes after therapy. Timing the therapeutic interventions based on the IFN activity and immune exhaustion dynamically may also improve the outcomes of therapy and ultimately the survival outcomes by enhancing the pathological response. The study of how NAT, the immune response, and tumor evolution intersect should be prioritized to develop precision oncology in EC. Rational combination approaches based on IFN-related immune exhaustion may eventually help overcome adaptive resistance, but their clinical benefit in EC must be confirmed in well-designed trials. At present, IFN-related markers should be viewed as promising exploratory biomarkers rather than established clinical decision-making tools. Future studies should validate these markers prospectively, distinguish ESCC from EAC, incorporate serial tumor sampling, and correlate molecular immune signatures with pathological response, recurrence, and survival.

## Glossary

CAV1: Caveolin-1
CRT: Chemoradiotherapy
CTLA-4: Cytotoxic T-lymphocyte–associated protein 4
DAMPs: Damage-associated molecular patterns
DDR: DNA damage response
EAC: Esophageal adenocarcinoma
EC: Esophageal cancer
ESCC: Esophageal squamous cell carcinoma
cGAS: Cyclic GMP-AMP synthase
GERD: Gastroesophageal reflux disease
ICB: Immune checkpoint blockade
ICIs: Immune checkpoint inhibitors
ICD: Immunogenic cell death
ICPs: Immune checkpoint pathways
ICRs: Immune checkpoint receptors
IER2: Immediate early response 2
IFN: Interferon
IFNGR1: Interferon gamma receptor 1
IL-10: Interleukin-10
IME: Immune-mediated exhaustion
IRGs: Interferon-related genes
ISGs: Interferon-stimulated genes
LAG-3: Lymphocyte activation gene-3
MDSCs: Myeloid-derived suppressor cells
MHC: Major histocompatibility complex
NAT: Neoadjuvant therapy
NK cells: Natural killer cells
PD-1: Programmed cell death protein 1
STING: Stimulator of interferon genes
TAMs: Tumor-associated macrophages
TGF-β: Transforming growth factor-β
TILs: Tumor-infiltrating lymphocytes
TIM-3: T-cell immunoglobulin and mucin-domain containing-3
TIME: Tumor immune microenvironment
TME: Tumor microenvironment
TMEM199: Transmembrane protein 199
Tregs: Regulatory T cells
VEGF: Vascular endothelial growth factor
